# Variation in the use of renal replacement therapy in patients with septic shock: a substudy of the prospective multicenter observational FINNAKI study

**DOI:** 10.1186/cc13716

**Published:** 2014-02-05

**Authors:** Meri Poukkanen, Juha Koskenkari, Suvi T Vaara, Ville Pettilä, Sari Karlsson, Anna-Maija Korhonen, Jouko J Laurila, Kirsi-Maija Kaukonen, Vesa Lund, Tero I Ala-Kokko

**Affiliations:** 1 Department of Anaesthesiology and Intensive Care, Lapland Central Hospital, Ounasrinteentie 22, 96440 Rovaniemi, Finland; 2 Department of Anaesthesiology, Division of Intensive Care, Oulu University Hospital and Medical Research Center Oulu, Kajaanintie 50, 90220 Oulu, Finland; 3 Intensive Care Unit, Division of Anaesthesia and Intensive Care Medicine, Department of Surgery, Helsinki University Central Hospital, Haartmaninkatu 4, 00029 Helsinki, Finland; 4 Department of Clinical Sciences, University of Helsinki, Tukholmankatu 8B, 00014 Helsinki, Finland; 5 Department of Intensive Care Medicine, Tampere University Hospital, Teiskontie 35, 33521 Tampere, Finland; 6 ANZIC-RC, Department of Epidemiology and Preventive Medicine, Monash University, 99 Commercial Road, Melbourne VIC 3004, Australia; 7 Department of Anaesthesia and Intensive Care Medicine, Satakunta Central Hospital, Sairaalantie 3, 28500 Pori, Finland

## Abstract

**Introduction:**

Indications for renal replacement therapy (RRT) have not been generally standardized and vary among intensive care units (ICUs). We aimed to assess the proportion, indications, and modality of RRT, as well as the association between the proportion of RRT use and 90-day mortality in patients with septic shock in Finnish adult ICUs.

**Methods:**

We identified patients with septic shock from the prospective observational multicenter FINNAKI study conducted between 1 September 2011 and 1 February 2012. We divided the ICUs into high-RRT and low-RRT ICUs according to the median of the proportion of RRT-treated patients with septic shock. Differences in indications, and modality of RRT between ICU groups were assessed. Finally, we performed an adjusted logistic regression analysis to evaluate the possible association of the ICU group (high vs. low-RRT) with 90-day mortality.

**Results:**

Of the 726 patients with septic shock, 131 (18.0%, 95% CI 15.2 to 20.9%) were treated with RRT. The proportion of RRT-treated patients varied from 3% up to 36% (median 19%) among ICUs. High-RRT ICUs included nine ICUs (354 patients) and low-RRT ICUs eight ICUs (372 patients). In the high-RRT ICUs patients with septic shock were older (*P* = 0.04), had more cardiovascular (*P* <0.001) and renal failures (*P* = 0.003) on the first day in the ICU, were more often mechanically ventilated, and received higher maximum doses of norepinephrine (0.25 μg/kg/min vs. 0.18 μg/kg/min, *P* <0.001) than in the low-RRT ICUs. No significant differences in indications for or modality of RRT existed between the ICU groups. The crude 90-day mortality rate for patients with septic shock was 36.2% (95% CI 31.1 to 41.3%) in the high-RRT ICUs compared to 33.9% (95% CI 29.0 to 38.8%) in the low-RRT ICUs, *P* = 0.5. In an adjusted logistic regression analysis the ICU group (high-RRT or low-RRT ICUs) was not associated with 90-day mortality.

**Conclusions:**

Patients with septic shock in ICUs with a high proportion of RRT had more severe organ dysfunctions and received more organ-supportive treatments. Importantly, the ICU group (high-RRT or low-RRT group) was not associated with 90-day mortality.

## Introduction

Sepsis is a common cause of acute kidney injury (AKI) [1,2] and up to 64% of patients with septic shock have a concomitant AKI [3-5]. According to previous studies 9 to 20% of the patients with septic shock receive renal replacement therapy (RRT) [3,6,7]. Septic AKI has a poor outcome [3,6-8] and it independently increases the risk of death [1].

The treatment of AKI is primarily supportive including RRT. Excluding few absolute indications for RRT, such as hyperkalemia, severe metabolic acidosis, overt uremia, and specific drug intoxications, the decision for the initiation of RRT is usually based on local clinical practice and the individual opinion of the attending physician. The Beginning and Ending Supportive Therapy for the Kidney (B.E.S.T) study reported marked practice variation for RRT globally [9]. Despite multiple studies, the optimal timing, modality, and anticoagulation of RRT are still largely unclear. According to recent studies even the overall beneficial effect of RRT is uncertain [10,11]. In patients with sepsis-associated AKI in a surgical intensive care unit (ICU) RRT has been found to be associated with increased mortality [10]. Likewise a recent study reported worse prognoses in patients with AKI receiving RRT compared to conservative treatment [11].

Accordingly, we aimed to assess the variation of RRT in patients with septic shock in this substudy of the multicenter prospective observational FINNAKI study [12]. Additionally, we evaluated the possible association of the relative proportion of RRT treatment (high- vs. low-RRT ICUs) and 90-day mortality in septic shock.

## Materials and methods

We retrieved patients with septic shock from the prospective, observational FINNAKI study conducted in 17 Finnish adults ICUs between 1 September 2011 and 1 February 2012 [12]. All emergency admissions and elective postoperative admissions with an expected ICU stay of more than 24 hours were included in the FINNAKI study. Intermediate care patients, patients on chronic dialysis, elective patients with an expected stay in the ICU of less than 24 hours, readmitted patients who had received RRT during the previous ICU admission, transferred patients who had already participated in the study for five days, patients with inadequate language skills or not permanently living in Finland, patients under 18 years of age, and organ donors were excluded from the study. The Ethics Committee of the Helsinki University Hospital approved the study protocol and the use of deferred consent (DNRO 18/13/03/02/1010). A written consent was obtained from patients or proxy.

### Definitions

We defined septic shock according to the American College of Chest Physicians/Society of Critical Care Medicine (ACCP/SCCM) criteria [13]. We used the Kidney Disease: Improving Global Outcome (KDIGO) criteria [14] to define and stage AKI by using both serum creatinine (SCr) and urine output criteria. KDIGO classification defines AKI as an increase in SCr by ≥26.5 μmol/l within 48 hours, or an increase in SCr to ≥1.5 times baseline, or urine volume less than 0.5 ml/kg/h for 6 hours. RRT-related complications were defined as follows: complication in catheter insertion (arterial insertion, pneumo- or hemothorax, severe hematoma), severe bleeding, hypotension during RRT (need for fluid resuscitation, increasing the dose of vasoactive treatment or discontinuing the treatment), severe electrolyte disturbance (hypophosphatemia, hypocalcemia, hypokalemia), and catheter-related infection. We defined organ failure as Sequential Organ Failure Assessment score (SOFA) ≥3 [15,16]. The probability of death was calculated according to the original Simplified Acute Physiology Score (SAPS) equation [17]. The data on survival at 90 days were obtained from the Finnish Population Register Center.

### Data source

For the current study we identified all patients with septic shock from the FINNAKI study [12]. The FINNAKI data were prospectively collected to the database of the Finnish Intensive Care Consortium maintained by Tieto Ltd., Helsinki, Finland. The database included demographic data, main physiologic and laboratory variables, SAPS II [17], SOFA scores [16], data on organ-supportive treatments, and outcomes. We used an additional internet-based case report form (CRF) to record data on chronic health status, medications, presence of severe sepsis or septic shock, and RRT. We collected CRF data daily for the first five days in the ICU and thereafter data on RRT twice a week of RRT-treated patients. Treatment restrictions were recorded as withholding or discontinuation of RRT, withdrawal of intensive care treatment, or decision not to resuscitate. The attending ICU physicians selected the indication for RRT from the following list: oliguria/anuria, azotemia/high creatinine, rhabdomyolysis, metabolic acidosis, hyperkalemia, fluid overload, immunomodulation, drug intoxication, and others. Multiple indications could be registered.

Altogether seventeen ICUs participated in the FINNAKI study (six university ICUs and eleven nonacademic, central hospital ICUs). Each participating ICU had the capacity to provide RRT. To assess the association of relative proportion of RRT use with 90-day mortality in patients with septic shock, we calculated the proportion of patients with septic shock treated with RRT in an individual ICU. The median of proportion of RRT was used as a cutoff value to divide the participating ICUs into two groups. The low-RRT group included eight ICUs (three university ICUs and five central hospital ICUs, altogether 372 patients) and the high-RRT group comprised nine ICUs (three university ICUs and six central hospital ICUs, altogether 354 patients).

### Statistics

We report continuous data as medians with interquartile range (IQR) and categorical data as absolute values and percentage. We report the main outcomes with 95% confidence interval (95% CI). We compared categorical data with chi-square or Fisher’s exact test and continuous data with Mann–Whitney *U* test. We calculated the standardized mortality ratio (SMR) by dividing the number of observed deaths with the predicted number of deaths according the original SAPS II equation [17]. To decrease the influence of treatment selection bias for initiation of RRT, we generated a propensity score by logistic regression [18,19]. In the propensity score we entered confounders related to the probability of receiving RRT: creatinine value on the first day in the ICU (D1), urine output on the D1, age categorized by Acute Physiology and Chronic Health Evaluation (APACHE) age groups [20], any comorbidity, SAPS II score without age and renal components, and SOFA score without renal points on the D1. We evaluated the association of the risk factors with 90-day mortality by univariable analysis. We then entered the factors with *P* <0.2 into the multivariable logistic regression model to analyze any possible association with 90-day mortality. We first performed the regression model for 90-day mortality without a propensity score and then with the propensity score excluding variables that interacted with the propensity score. We present the results of the logistic regression model with odds ratios (OR) with 95% CI. Goodness-of-fit was evaluated using the Hosmer-Lemeshow test. We report two-tailed *P* values and considered a *P* value less than 0.05 to be statistically significant. We performed all analyses using the IBM SPSS statistics software version 20.0 (IBM Corp., Armonk, NY, USA).

## Results

Altogether 726 patients fulfilled the criteria of septic shock. Of these 726 patients with septic shock, 131 (18.0%, 95% CI 15.2 to 20.9%) were treated with RRT. The main indications for RRT were oliguria (85%), acidosis (73.3%), high creatinine (60.3%), and fluid overload (42.0%). The data of the indication, modality, and anticoagulation of RRT are presented in the additional file (Table S1 in Additional file 1).

### Low-RRT and high-RRT ICUs

**Figure 1:**
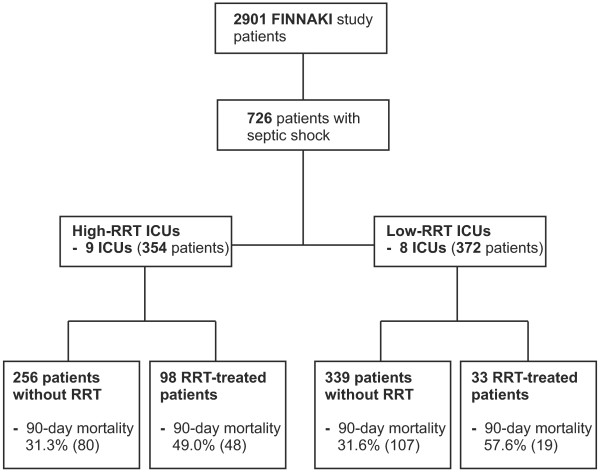
Flow chart of patients with septic shock with or without renal replacement therapy (RRT) in low-RRT and high-RRT intensive care units (ICUs).

**Figure 2:**
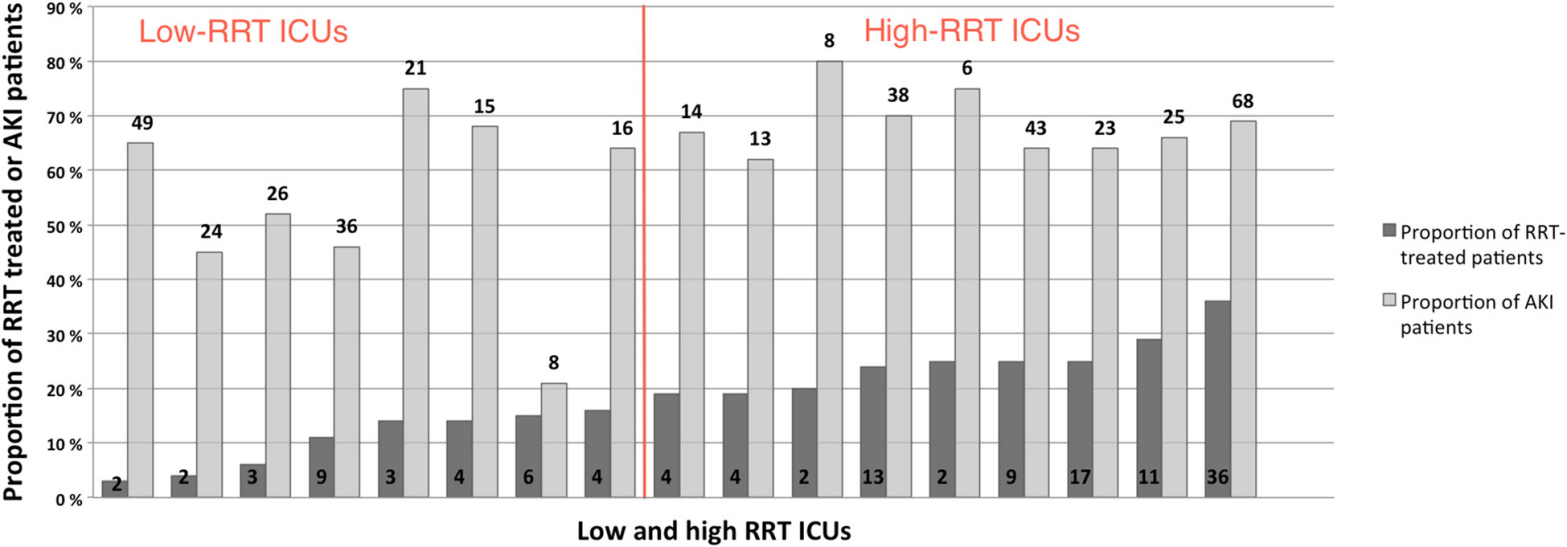
Proportions and absolute numbers of renal replacement therapy (RRT) delivered for patients and patients with acute kidney injury (AKI) in each intensive care unit (ICU).

The number of patients with septic shock in each ICU varied from 22 to 79 patients (median [IQR] 44.5 patients, [25.8 to 70.3]) in the low-RRT ICUs and from 8 to 99 patients (median [IQR] 36.0, [15.5 to 60.5]) in the high-RRT ICUs. Figure 1 presents the study flow chart with the number of patients with or without RRT in low- and high-RRT ICUs. The proportion of RRT-treated patients with septic shock ranged from 3% (2/76) to 16% (4/25) in the low-RRT ICUs and between 19% (4/21) and 36% (36/99) in the high-RRT ICUs (Figure 2). The proportions of patients with septic shock and AKI (that is, patients at risk for RRT) are presented in Figure 2. Of the participating six university ICUs, three belonged to the high-RRT ICUs (proportion of RRT 25%, 25%, and 35%) and three to the low-RRT ICUs (proportion of RRT 3%, 6%, and 11%). The median number of ICU beds in the low-RRT ICUs was 16.0 (8.0 to 19.0) and in the high-RRT ICUs 10.0 (7.0 to 16.0). The median [IQR] for total RRT-days in septic shock indexed to ICU beds was 2.1 [1.5 to 2.9] in the low-RRT ICUs compared to 4.5 [1.3 to 9.9] in the high-RRT ICUs, *P* = 0.2.

### Patients in the high-RRT and low-RRT ICUs

The patients with septic shock in the high-RRT ICUs were older (*P* = 0.04), had emergency (*P* <0.001), and operative (*P* = 0.01) admission more often, and had more abdominal infections (*P* = 0.007) than in the low-RRT ICUs (Table 1). The proportion of patients with AKI and the severity of AKI according to the KDIGO classification in the high- and low-RRT ICUs are presented in Table 2. The proportions of cardiovascular (*P* <0.001) and renal (*P* = 0.003) organ failures (SOFA 3 or 4) within the first day in the ICU were greater among patients treated in the high-RRT ICUs than in the low-RRT ICUs (Table 2). In the high-RRT ICUs patients were mechanically ventilated more often (*P* <0.001), received sepsis corticosteroid more often (*P* <0.001) and received a higher maximum dose of norepinephrine (*P* <0.001) during the first five days in the ICU (Table 2). The lowest base excess (BE) for 24 hours prior to ICU admission or within the first 24 hours in the ICU was -5.4 [-10.6 to (-2.5)] in the high-RRT ICUs compared to -4.1 [8.7 to (-1.1)] in the low-RRT ICUs, *P* <0.001. The highest lactate value during the first five days in the ICU was higher in the high-RRT ICUs [2.5 mmol/l, (1.6 to 4.4 mmol/l)] than in the low-RRT ICUs [2.2 mmol/l (1.4 to 4.0 mmol/l)], *P* = 0.02.

**Table 1:** Demographic of patients with septic shock divided by low- or high-renal replacement therapy (RRT) intensive care units (ICUs)

	**Patients in low-RRT ICUs (n = 372)**	**Patients in high-RRT ICUs (n = 354)**	***P *** **value**
Age (years)	64.0 [54.0–74.0]	67.0 [56.0–76.0]	0.04
Gender (male)	243 (65.3)	221 (62.4)	0.4
Any comorbidity^1^	237 (64.9)	250 (71.2)	0.07
Community-acquired infection	103 (29.5)	106 (32.8)	0.4
Source of infection			
Pulmonary	186 (54.4)	165 (52.1)	0.5
Abdominal	84 (24.6)	108 (34.1)	0.007
Genitourinary	21 (6.1)	26 (8.2)	0.3
Soft tissue	47 (13.7)	24 (7.6)	0.01
Emergency admission	370 (99.5)	334 (94.4)	<0.001
Operative admission	89 (23.9)	114 (32.2)	0.01
SAPS without age and renal components	27.0 [21.0–36.0]	26.0 [19.0–34.0]	0.02
SOFA D1	9.0 [7.0–11.0]	9.0 [7.0–11.0]	0.08
APACHE II diagnostic group			
Respiratory tract, nonoperative	92 (24.7)	82 (23.2)	0.6
Nonoperative sepsis	72 (19.4)	64 (18.1)	0.7
Gastrointestinal tract, operative	56 (15.1)	71 (20.1)	0.08
Gastrointestinal tract, nonoperative	32 (8.6)	30 (8.5)	0.9
Cardiovascular, nonoperative	30 (8.1)	31 (8.8)	0.7
Neurological, nonoperative	16 (4.3)	7 (2.0)	0.07
Metabolic	16 (4.3)	7 (2.0)	0.07
Trauma	12 (3.2)	5 (1.4)	0.1
Neurological, operative	11 (3.0)	2 (0.6)	0.02
Cardiovascular, operative	5 (1.3)	21 (5.9)	0.001
Treatment restrictions			
Any treatment restriction^2^	100 (26.9)	104 (29.4)	0.5
Withholding of RRT	28 (7.5)	16 (4.5)	0.09
Outcomes			
Length of stay ICU (days)	4.1 [2.1–8.1]	4.7 [2.7–8.3]	0.09
Length of stay hospital (days)	15.0 [8.0–25.0]	14.0 [7.0–24.0]	0.2
90-day mortality	126 (33.9)	128 (36.2)	0.5
Probability of death^3^	0.33 [0.17–0.62]	0.37 [0.17–0.64]	0.4
SMR (95% CI)	0.72 (0.6–0.84)	0.66 (0.58–0.74)	
Propensity for RRT^4^	0.06 [0.03–0.15]	0.06 [0.03–0.24]	0.01

Values are expressed as count (%) and median [interquartile range], except for SMR (with 95% confidence interval). ^1^Including COPD, chronic cardiovascular disease, diabetes, thromboembolic disease, chronic liver disease, vasculitis, organ transplant, cancer; ^2^including withholding and withdrawing of intensive care, withholding and withdrawal of RRT, and do not resuscitate restrictions; ^3^calculated from SAPS II score; ^4^confounders entered to the propensity score: creatinine value on the first day in the ICU (D1), urine output on the D1, age, any comorbidity, SAPS II score without age and renal components and SOFA score without renal points on the D1. SOFA D1, Sequential Organ Failure Assessment on the first day in the ICU; APACHE II, Acute Physiology and Chronic Health Evaluation; SAPS II, Simplified Acute Physiology Score II; AKI, acute kidney injury; KDIGO, Kidney Disease: Improving Global Outcomes; SMR, standardized mortality ratio.

**Table 2:** Comparison of organ failures and organ-supportive treatments between high- and low-renal replacement therapy (RRT) intensive care units (ICUs)

	**Low RRT-ICUs (n = 372)**	**High RRT-ICUs (n = 354)**	***P *** **value**
Septic shock on ICU admission	109 (29.3)	138 (39.0)	0.006
Any AKI	196 (52.7)	240 (67.8)	<0.001
KDIGO stage 1	85 (22.8)	82 (23.2)	0.9
KDIGO stage 2	41 (11.0)	43 (12.1)	0.6
KDIGO stage 3	70 (18.8)	115 (32.5)	<0.001
KDIGO 3 stage without RRT	37 (9.9)	17 (4.8)	0.008
Cardiovascular failure on D1	317 (85.2)	336 (94.9)	<0.001
Respiratory failure on D1	193 (51.9)	179 (50.6)	0.7
Renal failure on D1	53 (14.2)	81 (22.9)	0.003
Liver failure on D1	4 (1.1)	8 (2.3)	0.2
Coagulation failure on D1	24 (6.5)	33 (9.3)	0.2
Central nervous system failure on D1	89 (23.9)	62 (17.5)	0.03
Number of organ failures during ICU stay	2.0 [2.0–3.0]	2.0 [2.0–3.0]	0.8
0–1 organ failure	81 (21.8)	84 (23.7)	0.5
2 organ failures	149 (40.1)	138 (39.0)	0.8
3–4 organ failures	139 (37.4)	112 (31.6)	0.1
5–6 organ failures	3 (0.8)	20 (5.6)	<0.001
Supportive treatments			
Mechanical ventilation	276 (74.2)	297 (83.9)	<0.001
Sepsis corticosteroid	99 (27.0)	138 (40.0)	<0.001
RRT	33 (8.9)	98 (27.7)	<0.001
Maximum dose of norepinephrine during the first five days in the ICU (μg/kg/min)	0.18 [0.08–0.38]	0.25 [0.13–0.67]	<0.001
Received furosemide	262 (70.4)	285 (80.5)	0.002

Values are expressed as count (%) and median [interquartile range]. AKI, acute kidney injury; KDIGO, Kidney Disease: Improving Global Outcomes; D1, first day in the ICU.

### RRT treatment

Patients treated with RRT did not differ in age, gender, or severity of illness between high- and low-RRT ICUs (Additional file 2). Apart from high creatinine, indications for and modalities of RRT were corresponding in both ICU groups. RRT was initiated in both ICU groups within the first 24 hours in the ICU. Citrate was used more often in the low-RRT ICUs than in the high-RRT ICUs (*P* = 0.004), otherwise the use of anticoagulation for RRT was similar in ICU groups. Table 3 presents the data of RRT in high- and low-RRT ICUs. The laboratory values preceding RRT did not differ between high- and low-RRT groups (Additional file 3). Patients without RRT were older in the high-RRT ICUs, and they received more often furosemide than in the low-RRT ICUs (Additional file 4).

**Table 3:** Treatment indication, modality, and anticoagulation of renal replacement therapy (RRT) in the low-RRT and high-RRT intensive care units (ICUs)

	**Low-RRT ICUs (n = 33)**	**High-RRT ICUs (n = 98)**	***P *** **value**
Indication of RRT			
Oliguria	29 (87.9)	83 (84.7)	0.7
High creatinine	25 (75.8)	54 (55.1)	0.04
Acidosis	24 (72.7)	72 (73.5)	0.9
Hyperkalemia	9 (27.3)	20 (20.4)	0.4
Fluid overload	12 (36.4)	43 (43.9)	0.4
Intoxication	2 (6.1)	3 (3.0)	0.4
Modality of RRT			
Only CRRT during ICU	20 (60.6)	52 (53.1)	0.5
Only IRRT during ICU	2 (6.1)	11 (11.2)	0.4
Both CRRT + IRRT during ICU	11 (33.3)	35 (35.7)	0.8
Time to initiation of RRT from ICU admission (hours)	17.8 [5.2–33.7]	13.5 [5.2–33.2]	0.9
Received anticoagulation	32 (97)	83 (84.7)	0.06
Citrate	23 (69.7)	40 (40.8)	0.004
LMWH	18 (54.5)	65 (66.3)	0.2
Other	0	7 (7.1)	0.1
None	4 (12.1)	27 (27.6)	0.07
Complications related to RRT			
Complication in insertion of catheter	8 (24.2)	10 (10.2)	0.04
Electrolyte disturbances	7 (21.2)	19 (19.4)	0.8
Hypotension during RRT	1 (3.0)	2 (2.0)	0.7
Bleeding	2 (6.1)	1 (1.0)	0.09
Catheter-related infection	0	1 (1.0)	0.6

Values are expressed as count (%) and median [interquartile range]. CRRT, continuous renal replacement therapy; IRRT, intermittent renal replacement therapy; LMWH, low-molecular-weight heparin.

### Treatment restrictions

Of the 726 patients with septic shock, RRT was restricted in 71 patients (9.8%) comprising 44 (6.1%) withholdings and 27 (3.7%) withdrawals of RRT. Patients with restricted RRT were older (69.4 vs. 65.0 years, *P* = 0.009), had higher nonrenal SOFA score on the first day in ICU (11.0 vs. 9.0, *P* = 0.004) and higher SAPS II score on admission (59.5 vs. 44.0, *P* <0.001) than patients without RRT restrictions. Any treatment restriction (withdrawal of intensive care, withholding or withdrawal of RRT, and decision not to resuscitate) was made as often in the low-RRT group as in the high-RRT group (Table 1). There were no differences in age, number of comorbidities, nonrenal SOFA score on the first day in ICU, or SAPS II score in patients with restricted initiation of RRT between ICU groups (data not shown). For RRT-treated patients treatment restrictions were made in 57/131 (43.5%) cases compared to 147/595 (24.7%) in patients without RRT, *P* <0.001.

### 90-day mortality

The crude 90-day mortality rates of patients with septic shock did not differ between the high-RRT and low-RRT ICUs (Table 1). Likewise, there was no significant difference in the crude 90-day mortality in RRT-treated patients with septic shock between high-RRT (49.0%, 95% CI 38.8 to 59.1%), and low-RRT ICUs (57.6%, 95% CI 40.4 to 74.5%), *P* = 0.39 (Figure 1). The probability of death and SMRs of patients with septic shock or those treated with RRT between the ICU groups did not differ (Table 1 and Additional file 2). Of the 71 patients with restricted RRT (withhold or withdrawal), 60 (84.5%) died within 90 days. In an adjusted multivariate logistic regression model the group of ICU (high- or low-RRT ICUs) and use of RRT were not associated with 90-day mortality and the result remained after adjustment with propensity score of RRT (Table 4).

**Table 4:** Results of univariable and multivariable logistic regression analyses for factors associated with 90-day mortality in patients with septic shock

	**Univariable analyses**		**Multivariable analyses**		**Propensity score**^**4 **^**adjusted multivariable analyses**	
	**OR (95% CI)**	***P***	**OR (95% CI)**	***P***	**OR (95% CI)**	***P***
Age					Not included	
45–54 years^1^	1.54 (0.7–3.37)	0.28	1.76 (0.73–4.28)	0.21		
55–64 years^1^	1.9 (0.94–3.82)	0.08	2.09 (0.95–4.6)	0.07		
65–74 years^1^	4.06 (2.06–8.02)	<0.001	5.52 (2.57–11.89)	<0.001		
≥75 years^1^	5.62 (2.86–11.04)	<0.001	8.26 (3.83–17.81)	<0.001		
Operative admission	0.57 (0.4–0.81)	0.002	0.63 (0.42–0.96)	0.03	0.6 (0.4–0.89)	0.01
SAPS II without age and renal points	1.05 (1.04–1.07)	<0.001	1.05 (1.03–1.07)	<0.001	Not included	
Renal failure within the first day on the ICUs	3.17 (2.16–4.66)	<0.001	1.91 (1.1–3.31)	0.02	Not included	
Mechanical ventilation	1.91 (1.27–2.87)	0.002	1.26 (0.76–2.09)	0.38	1.77 (1.12–2.77)	0.01
Highest norepinephrine dose (μg/kg/min)^2^	2.74 (1.92–3.91)	<0.001	1.02 (1.01–1.03)	<0.001	1.02 (1.01–1.02)	<0.001
Lowest BE value^3^	0.94 (0.92–0.96)	<0.001	0.98 (0.95–1.01)	0.11	0.97 (0.94–1.0)	0.05
High RRT ICU group	1.11 (0.82–1.5)	0.52	0.89 (0.59–1.34)	0.57	0.88 (0.6–1.29)	0.50
RRT*RRT group (interaction term)	1.97 (1.28–3.02)	0.02	0.67 (0.24–1.85)	0.43	0.7 (0.27–1.79)	0.45

^1^Compared to patients under 44 years of age; ^2^the highest norepinephrine dose during the first five days in the ICU; ^3^the lowest BE value 24 hours prior to ICU admission or within the first 24 hours in the ICU; ^4^confounders entered to the propensity score: creatinine value on the first day in the ICU (D1), urine output on the D1, age groups according to APACHE II score, any comorbidity, SAPS II score without age and renal components and SOFA score without renal points on the D1. OR odds ratio; CI, confidence interval; SAPS II, Simplified Acute Physiology Score II; RRT, renal replacement therapy; ICU, intensive care unit.

Multivariable model without the propensity score: Hosmer-Lemeshow *P* = 0.59 and -2 log likelihood = 738.8. Multivariable model with the propensity score: Hosmer-Lemeshow *P* = 0.46 and -2 log likelihood =800.2.

## Discussion

In this prospective multicenter study we found a 10-fold variation (3% to 36%) in the proportion of RRT among patients with septic shock across Finnish ICUs. There were significant differences in case-mix and severity of organ dysfunctions between the high- and low-RRT ICUs, but indications for and modality of RRT were comparable. Despite the variation in proportion of RRT the 90-day mortality rates for patients with septic shock or RRT-treated patients with septic shock did not differ between the ICU groups.

### Absolute and proportional RRT volume in patients with septic shock

In the present study 18% of patients with septic shock received RRT. Our finding is in concordance with a recent French study [6] but other studies have reported much higher proportions of RRT use in this group of patients varying from 30% up to 71% [3,7,21].

The differences in case-mix between low and high-RRT ICUs explained the variation in the proportion of RRT-treated patients. Although SAPS II score and SOFA score did not differ between ICU groups, patients in the high-RRT ICUs were more severely ill in terms of presences of septic shock on ICU admission, number of patients with renal failure (SOFA 3 to 4) within the first day in the ICUs, and number of patients with at least five organ failures. In addition to higher prevalence of RRT in septic shock, these patients also received other organ-supportive treatments more often, such as mechanical ventilation, sepsis corticosteroid treatment, and higher maximum doses of norepinephrine during the first five days in the ICU in the high-RRT ICUs. Likewise the number of patients at risk for RRT (patients with AKI KDIGO stage 1 to 3) was significantly higher in the high-RRT ICU group.

### Indications, modality, and complications of RRT

The national guideline for the treatment of AKI was published in Finland in 2009 [22], which may explain the high rate of corresponding indications for and modalities of RRT between high-RRT and low-RRT ICUs in the treatment of patients with septic shock. In addition, the main indications for RRT in both ICU groups were in concordance with the B.E.S.T. study [9]. Likewise, the frequencies of complications of RRT, except for catheter insertion complications, did not differ between high-RRT and low-RRT ICUs.

### Treatment restrictions and 90-day mortality

In the present study treatment restrictions were made in a quarter of patients with septic shock without RRT compared to nearly 45% of RRT-treated patients. The high-RRT and the low-RRT ICUs did not differ in frequency of restriction of RRT and there were no differences in age, number of comorbidities or severity of illness in patients with restricted initiation of RRT between ICU groups. Severity of illness, poor prognosis, and age has been shown to associate with treatment restrictions in patients with AKI [23]. In our study, however, the withdrawal from RRT treatment was more frequent in the low-RRT ICUs. Likewise, AKI has been reported to be associated with a higher rate of withdrawal of intensive care treatment than with other supportive treatments [24]. In a study of hypothetical patient cases physicians were more likely to withhold or withdraw dialysis or mechanical ventilation than other treatments [25].

The 90-day mortality rates did not differ between ICU groups regardless of the substantial variation in the proportion of RRT-treated patients with septic shock. Moreover, in an adjusted logistic regression model the group of ICU (low- or high-RRT ICUs) was also not associated with mortality. This result was supported by the equal SMR values between low- and high-RRT ICUs. Taken together, our finding suggests that resource utilization during the study period was equal with corresponding patient selection for RRT treatment. In contrast to other recent studies [10,11,26], the use of RRT was not associated with 90-day mortality in the present study.

### Limitations

The strengths of our study are the prospective data collection and multicenter study design covering the majority of the adult population in Finland [12]. Our study does, however, have some limitations. First, the relatively small number of RRT-treated patients with septic shock in both ICU groups makes it difficult to draw robust conclusions whether these patients or indications for RRT were similar in the low- and high-RRT ICUs. Also the study period (five months) may have influenced the proportion of RRT-treated patients in each ICU due to seasonal alterations in the number of septic shock patients needing RRT treatment. We believe, however, that our finding reflects the genuine practice of RRT in septic shock across Finnish ICUs. Second, our definition of high- and low-RRT ICUs may be considered arbitrary. The relatively small number of patients with septic shock with only a few RRT-treated patients would give a high proportion of RRT. Also the units we classified as high-RRT ICUs may be considered as low elsewhere according to the absolute number of cases. However, instead of evaluating the impact of the size of ICU, we attempted to assess the association of administration of RRT treatment with the 90-day mortality in patients with septic shock. Third, the most severely ill patients (for example severe trauma and patients requiring neurosurgery or cardiac surgery) were transferred to university ICUs for special treatment. This may have decreased the proportion of RRT-treated patients in the transferring ICU. University and central hospital ICUs were, however, equally represented in the high- and low-RRT ICUs. Fourth, we evaluated only patients treated in the ICU. Finally, since no global guidelines on the initiation of or indication for RRT exist, treatment selection bias may have influenced our results. Although we generated the propensity score for RRT to decrease the treatment selection bias, some significant confounders may be missing from the analysis. Likewise as our study was an observational study, we cannot determine whether the indication for RRT or absence of RRT was relevant. This may also under- or overestimate the proportion of RRT-treated patients in each ICU.

## Conclusions

Patients with septic shock in ICUs with a high proportion of RRT had more severe organ dysfunctions and received more organ-supportive treatments. The ICU group (high-RRT or low-RRT group) was not associated with 90-day mortality.

## Key messages

• Differences in case mix and severity of organ dysfunctions in patients with septic shock across Finnish ICUs explained the 10-fold variation in the proportion of RRT-treated patients.

• The indications for and modality of RRT were mainly corresponding between high- and low-RRT ICUs.

• The crude 90-day mortality rates as well as the standardized mortality ratios did not differ between high-RRT and low-RRT ICUs.

• In adjusted logistic regression analysis the ICU group (high- or low-RRT group) was not associated with 90-day mortality.

## Abbreviations

ACCP/SCCM: American College of Chest Physicians/Society of Critical Care Medicine; AKI: acute kidney injury; APACHE: Acute Physiology and Chronic Health Evaluation; BE: base excess; CI: confidence interval; CRF: case report form; CRRT: continuous renal replacement therapy; D1: first day in the ICU; ICD-10: International Classification of Diseases; ICU: intensive care unit; IQR: interquartile range; IRRT: intermittent renal replacement therapy; KDIGO: Kidney Disease: Improving Global Outcomes; LMWH: low-molecular-weight heparin; MDRD: modification in diet in renal disease; OR: odds ratio; RRT: renal replacement therapy; SAPS II: Simplified Acute Physiology Score II; SCr: serum creatinine; SMR: standardized mortality ratio; SOFA: Sequential Organ Failure Assessment; UFH: unfractionated heparin.

## Competing interests

The authors declare they have no competing interests.

## Authors’ contributions

MP participated in the design and data gathering of the study and performed the data analysis and drafted the manuscript. JK participated in the design and data gathering of the study and critically revised the manuscript. STV participated in the data gathering of the study and helped to perform the statistical analysis, and to draft the manuscript. VP participated in the design and coordination of the study and helped to draft the manuscript. SK, AMK, and, KMK participated in the design and coordination of the study and critically revised the manuscript. JJL and VL participated in data gathering and critically revised the manuscript. TAK participated in designing the study, helped to perform the statistical analysis and to draft the manuscript. All authors read and approved the final manuscript.

## Supplementary Material

Additional file 1: Table S1 Indication for and modality of renal replacement therapy (RRT) and the use of anticoagulation during RRT in patients with septic shock. Click here for file

Additional file 2: Table S2 Demographic data and treatment restrictions of renal replacement therapy (RRT)-treated patients with septic shock in low-RRT and high-RRT ICUs. Click here for file

Additional file 3: Table S3 Laboratory values prior to initiation of renal replacement therapy (RRT) divided in low-and high-RRT ICUs. Click here for file

Additional file 4: Table S4 Data of patients with septic shock without renal replacement therapy (RRT) treatment. Click here for file

## The FINNAKI study group

*Central Finland Central Hospital:* Raili Laru-Sompa, Anni Pulkkinen, Minna Saarelainen, Mikko Reilama, Sinikka Tolmunen, Ulla Rantalainen, Marja Miettinen. *East Savo Central Hospital:* Markku Suvela, Katrine Pesola, Pekka Saastamoinen, Sirpa Kauppinen. *Helsinki University Central Hospital:* Ville Pettilä, Kirsi-Maija Kaukonen, Anna-Maija Korhonen, Sara Nisula, Suvi Vaara, Raili Suojaranta-Ylinen, Leena Mildh, Mikko Haapio, Laura Nurminen, Sari Sutinen, Leena Pettilä, Helinä Laitinen, Heidi Syrjä, Kirsi Henttonen, Elina Lappi, Hillevi Boman. *Jorvi Central Hospital:* Tero Varpula, Päivi Porkka, Mirka Sivula Mira Rahkonen, Anne Tsurkka, Taina Nieminen, Niina Prittinen. *Kanta*-*Häme Central Hospital:* Ari Alaspää, Ville Salanto, Hanna Juntunen, Teija Sanisalo. *Kuopio University Hospital:* Ilkka Parviainen, Ari Uusaro, Esko Ruokonen, Stepani Bendel, Niina Rissanen, Maarit Lång, Sari Rahikainen, Saija Rissanen, Merja Ahonen, Elina Halonen, Eija Vaskelainen. *Lapland Central Hospital:* Meri Poukkanen, Esa Lintula, Sirpa Suominen*. Länsi Pohja Central Hospital:* Jorma Heikkinen, Timo Lavander, Kirsi Heinonen, Anne-Mari Juopperi. *Middle Ostrobothnia Central Hospital:* Tadeusz Kaminski, Fiia Gäddnäs, Tuija Kuusela, Jane Roiko. *North Karelia Central Hospital:* Sari Karlsson, Matti Reinikainen, Tero Surakka, Helena Jyrkönen, Tanja Eiserbeck, Jaana Kallinen. *Satakunta Hospital District:* Vesa Lund, Päivi Tuominen, Pauliina Perkola, Riikka Tuominen, Marika Hietaranta, Satu Johansson. *South Karelia Central Hospital:* Seppo Hovilehto, Anne Kirsi, Pekka Tiainen, Tuija Myllärinen, Pirjo Leino, Anne Toropainen. *Tampere University Hospital:* Anne Kuitunen, Ilona Leppänen, Markus Levoranta, Sanna Hoppu, Jukka Sauranen, Jyrki Tenhunen, Atte Kukkurainen, Samuli Kortelainen, Simo Varila. *Turku University Hospital:* Outi Inkinen, Niina Koivuviita, Jutta Kotamäki, Anu Laine*. Oulu University Hospital:* Tero Ala-Kokko, Jouko Laurila, Sinikka Sälkiö. *Vaasa Central Hospital:* Simo-Pekka Koivisto, Raku Hautamäki, Maria Skinnar.
