# Supplementary material for: Variation in the use of renal replacement therapy in patients with septic shock: a substudy of the prospective multicenter observational FINNAKI study
Source: Crit Care. 2014 Feb 5;18(1):R26. doi: 10.1186/cc13716 (PMC4056326; doi:10.1186/cc13716)
Supplement: Additional file 1: Table S1 — Indication for and modality of renal replacement therapy (RRT) and the use of anticoagulation during RRT in patients with septic shock. [file cc13716-S1.docx]

Additional file 1. Table S1 Indication and modality of renal replacement therapy (RRT) and the use of anticoagulation during RRT in patients with septic shock.

|  | RRT-treated patients (N=131) |
| --- | --- |
| Indication of RRT | |
| Oliguria | 112 (85) |
| Acidosis | 96 (73.3) |
| High creatinine | 79 (60.3) |
| Fluid overload | 55 (42.0) |
| Other | 32 (24.4) |
| Else | 5 (3.8) |
| Modality of RRT | |
| Only CRRT during ICU stay | 72 (55.0) |
| Only IRRT during ICU stay | 13 (9.9) |
| CRRT and IRRT during ICU stay | 46 (35.1) |
| Anticoagulation for RRT | 115 (87.8) |

Values are expressed as count (%).

Other Immunomodulation or intoxication, CRRT continuous renal replacement therapy, IRRT intermittent renal replacement therapy, ICU intensive care unit
