# Supplementary material for: Variation in the use of renal replacement therapy in patients with septic shock: a substudy of the prospective multicenter observational FINNAKI study
Source: Crit Care. 2014 Feb 5;18(1):R26. doi: 10.1186/cc13716 (PMC4056326; doi:10.1186/cc13716)
Supplement: Additional file 2: Table S2 — Demographic data and treatment restrictions of renal replacement therapy (RRT)-treated patients with septic shock in low-RRT and high-RRT ICUs. [file cc13716-S2.docx]

Additional file 2. Table S2 Demographic data and treatment restrictions of RRT-treated patients with septic shock in low-RRT and high-RRT ICUs

|  | Patients in  low-RRT ICUs (n=33) | Patients in  high-RRT ICUs (n=98) | P-value |
| --- | --- | --- | --- |
| Age (median) | 63.0 [55.5-75.0] | 65.0 [55.0-75.0] | 0.9 |
| Gender (male) | 22 (57.9) | 61 (62.3) | 0.4 |
| Any co-morbidity | 24 (72.2) | 72 (73.5) | 0.9 |
| SAPS II | 63.0 [50.5-71.5] | 56.0 [5.0-68.3] | 0.2 |
| SOFA D1 | 13.0 [10.0-14.0] | 11.5 [10.0-15.0] | 0.9 |
| Any treatment restrictions | 17 (51.5) | 40 (40.8) | 0.3 |
| RRT discontinued | 11 (33.3) | 16 (16.3) | 0.04 |
| Withdrawal of intensive care | 15 (45.5) | 35 (35.7) | 0.3 |
| Length of stay (days) | | | |
| ICU | 7.7 [2.2-15.4] | 6.9 [3.9-11.9] | 0.9 |
| Probability of death ^1^ | 0.74 [0.47-0.86] | 0.60 [0.37-0.82] | 0.23 |
| SMR (95%CI) | 0.80 (0.62-0.97) | 0.72 (0.6-0.83) |  |

Values are expressed as count (%) and median [interquartile range] except for SMR (with 95% confidential interval). ^1^ Calculated from SAPS II score. RRT renal replacement therapy, ICU intensive care unit, SOFA D1 Sequential Organ Failure Assessment on the first day in the ICU, SAPS II Simplified Acute Physiology Score, SMR Standardized mortality ratio
