# Supplementary material for: Variation in the use of renal replacement therapy in patients with septic shock: a substudy of the prospective multicenter observational FINNAKI study
Source: Crit Care. 2014 Feb 5;18(1):R26. doi: 10.1186/cc13716 (PMC4056326; doi:10.1186/cc13716)
Supplement: Additional file 3: Table S3 — Laboratory values prior to initiation of renal replacement therapy (RRT) divided in low-and high-RRT ICUs. [file cc13716-S3.docx]

Additional file 3. Table S3. Laboratory values of prior to initiation of renal replacement treatment (RRT) divided in low- and high-RRT ICUs

|  | Low-RRT ICUs | High-RRT ICUs | P-value |
| --- | --- | --- | --- |
| pH | 7.28 (7.18-7.35) | 7.3 (7.2-7.4) | 0.7 |
| BE | -9.5 [-12.6- (-5.7)] | -10.0 [-14.4- (-5.8)] | 0.7 |
| Lactate (mmol/L) | 3.0 (1.7-4.5) | 3.3 (1.4 -8.6) | 0.6 |
| Creatinine (µmol/L) | 293.0 (189.5-411.5) | 214.0 (129.0-388.0) | 0.1 |
| Fluid overload (L) | 4.6 (2.4-8.8) | 6.4 (2.4-9.8) | 0.3 |
| Urea (mmol/L) | 23.8 (13.3-30.7) | 22.5 (17.3-34.1) | 0.5 |

Values are expressed as median (IQR)

RRT renal replacement therapy, ICU intensive care unit, BE base excess
