# Supplementary material for: Variation in the use of renal replacement therapy in patients with septic shock: a substudy of the prospective multicenter observational FINNAKI study
Source: Crit Care. 2014 Feb 5;18(1):R26. doi: 10.1186/cc13716 (PMC4056326; doi:10.1186/cc13716)
Supplement: Additional file 4: Table S4 — Data of patients with septic shock without renal replacement therapy (RRT) treatment. [file cc13716-S4.docx]

Additional file 4. Table S4. Data of patients with septic shock without RRT-treatment

|  | Low-RRT (N=339) | High-RRT (n=256) | p-value |
| --- | --- | --- | --- |
| Age (years) | 64.0 [53.0-74.0] | 68.0 [56.0-77.0] | 0.02 |
| SAPS II (points) | 43.0 [34.0-55.0] | 43.0 [33.0-54.0) | 0.4 |
| SOFA renal 3-4 (points) | 40 (11.8%) | 25 (9.8) | 0.4 |
| Any AKI | 163 (48.1) | 142 (55.5) | 0.07 |
| KDIGO stage 3 | 37 (10.9) | 17 (6.6) | 0.07 |
| RRT restricted | 25 (7.4) | 19 (7.4) | 0.9 |
| Any treatment restriction | 83 (24.5) | 64 (25.0) | 0.9 |
| Use of furosemide | 240 (70.8) | 219 (85.5) | <0.001 |
| 90d mortality | 107 (31.6) | 80 (31.3) | 0.9 |

Values are expressed as median (interquartile range) and count (%)

RRT renal replacement therapy, SAPS II Simplified Acute Physiology Score, SOFA Sequential Organ Failure Assessment, AKI acute kidney injury
